# Comparison of K-TIRADS, EU-TIRADS and ACR-TIRADS Guidelines for Malignancy Risk Determination of Thyroid Nodules

**DOI:** 10.3390/diagnostics15081015

**Published:** 2025-04-16

**Authors:** Eren Tobcu, Erdal Karavaş, Gülden Taşova Yılmaz, Bilgin Topçu

**Affiliations:** 1 Department of Radiology, Bandırma Onyedi Eylul University School of Medicine, 10200 Balıkesir, Türkiye; ekaravas@bandirma.edu.tr (E.K.); btopcu@bandirma.edu.tr (B.T.); 2 Department of Pathology, Bandırma Research and Training Hospital, 10200 Balıkesir, Türkiye; gtasova@hotmail.com

**Keywords:** thyroid nodule, thyroid imaging reporting and data system (TIRADS), fine-needle aspiration biopsy (FNAB)

## Abstract

**Background/Objectives**: Thyroid nodules are commonly observed in neck ultrasonography. Most nodules are benign; hence, several nodules require biopsy to accurately identify the malignant ones. Numerous risk classification guidelines have been developed for thyroid nodules, varying in their indications for fine-needle aspiration biopsy (FNAB). The aim of this study is to evaluate the performances of three internationally recognized thyroid imaging reporting and data systems (TIRADS) for risk stratification of malignancy in comparison to one another. **Methods**: A total of 225 thyroid nodules with definitive FNAB cytology or histopathological diagnoses were included in this study. Various ultrasound (US) features were classified into categories based on three TIRADS editions. The guidelines were assessed regarding sensitivity, specificity, predictive values, and diagnostic accuracy to compare diagnostic value. **Results**: The American College of Radiology (ACR)-TIRADS demonstrated the best diagnostic accuracy (63.1%), the highest specificity (58.7%) and positive predictive value (36.3%), among three different TIRADS systems. Korean (K)-TIRADS exhibited the highest sensitivity (94.2%), negative predictive value (96.1%), and the most favorable negative likelihood ratio (0.13). The European (EU)-TIRADS had a sensitivity of 90.4%, specificity of 48.6%, and diagnostic accuracy of 58.2%, ranking between the other two guidelines across most parameters. **Conclusions**: The rigorous use of the guidelines established by each of the three TIRADS systems would have markedly reduced the number of FNABs performed. The comparison of the three guidelines in our study indicated that they are effective screening methods for identifying malignant thyroid nodules. Among them, K-TIRADS showed the most effective diagnostic performance in sensitivity, while ACR-TIRADS yielded the best specificity.

## 1. Introduction

The prevalence of thyroid nodules has been rising in recent years due to the widespread utilization of ultrasonography (US). The reported prevalence changes up to 68% of patients who undergo high-frequency US examination [1,2]. When a new nodule is detected in the thyroid gland, the primary goal is to discriminate the malignant nodules from benign ones [3,4]. Nevertheless, a significant number of patients with malignant nodules do not exhibit any distinct clinical signs or laboratory abnormalities prior to the occurrence of distant metastases. This poses a challenge in distinguishing between malignant and benign nodules [5,6,7,8]. Fine-needle aspiration biopsy (FNAB) is the main diagnostic method that is used for this reason [9,10,11]. Nevertheless, a minimum of 50% of all biopsied nodules are determined to be benign, and around 33% have cytological results that are uncertain [12,13].

Since the late 1990s, numerous studies have been performed to examine the relationship between the specific sonographic characteristics of thyroid nodules and the presence of malignancy. These sonographic characteristics are also utilized in the decision-making process for FNAB [14,15,16,17]. However, none of those individual parameters effectively predict the probability of malignancy [18,19]. In 2009, Horvath et al. developed the initial thyroid imaging reporting and data system (TIRADS) to increase the accuracy of diagnosing thyroid nodules using US features [20]. In 2015, 2016 and 2017, the American College of Radiology, the Korean Society of Thyroid Radiology and the European Thyroid Association separately released guidelines known as ACR-TIRADS, K-TIRADS and EU-TIRADS. The latest versions of these guidelines were released in 2017, 2021 and 2023, respectively [8,21,22]. However, while several studies have compared the efficacy of these risk stratification systems [23,24,25], their effectiveness in different nodule subtypes still requires further evaluation.

The aim of our study was to evaluate the diagnostic accuracy of the ACR-TIRADS, K-TIRADS and EU-TIRADS classification systems in characterizing nodules and to assess the rates of unnecessary FNABs.

## 2. Materials and Methods

This prospective study was conducted in the Radiology Department of an academic referral center. Patients who were referred to our department for FNAB of a thyroid nodule between 1 April 2024 and 31 December 2024 were included in the study. Patients with previously diagnosed thyroid malignancy were excluded from the study. The study was performed in accordance with the ethical guidelines of the Helsinki Declaration and approved by the local ethics review committee (2024-3).

**Figure 1:**
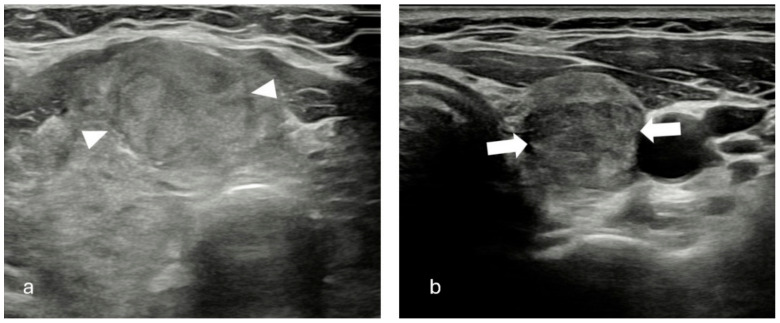
(**a**) Solid, iso/hyperechoic nodule with smooth margins (arrow heads). TR-3, EU-TIRADS 2, K-TIRADS 3. Histopathology: Benign thyroid nodule. (**b**) Solid, hypoechoic nodule with smooth margins (white arrows). TR-4, EU-TIRADS 4, K-TIRADS 4. Histopathology: Benign thyroid nodule.

**Figure 2:**
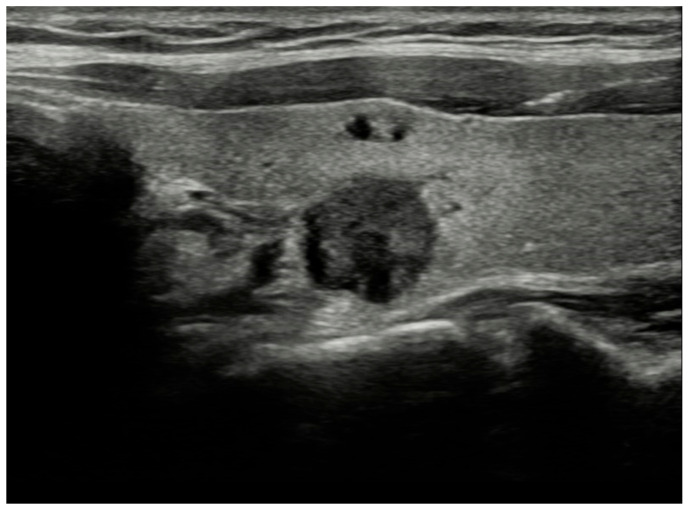
Solid, very hypoechoic nodule with irregular/lobulated margin. TR-5, EU-TIRADS 5, K-TIRADS-5. Histopathology: papillary thyroid cancer.

**Figure 3:**
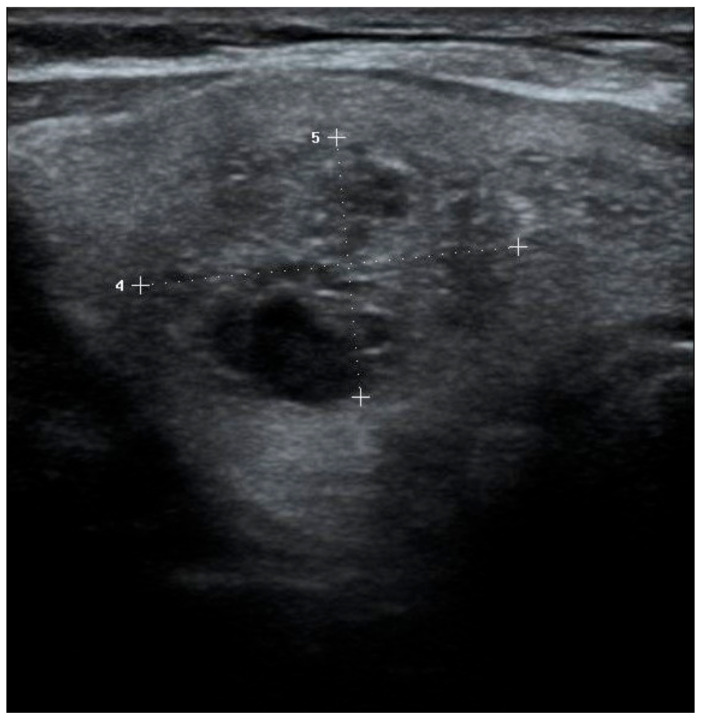
Solid, isoechoic nodule with punctate echogenic foci. TR-4, EU-TIRADS 5, K-TIRADS-4. Histopathology: Papillary thyroid cancer.

Two senior radiologists, one with 13 years of experience and another with 20 years of experience in ultrasound, conducted all examinations utilizing RS85 ultrasound devices from Samsung Healthcare. These devices were equipped with LA2-14A (2–14 MHz) linear transducers. The patients underwent US examinations in supine position with their neck slightly extended. Observers documented their assessment regarding the sonographic characteristics of each nodule with consensus. Each nodule was evaluated for its dimensions, composition, echogenicity, shape, margin, and presence of echogenic foci. Nodule composition was classified as cystic or almost completely cystic, spongiform, mixed cystic and solid, solid or almost completely solid. Nodule echogenicity was categorized as anechoic (for cystic or almost cystic nodules), hyperechoic/isoechoic/hypoechoic (compared to adjacent parenchyma), very hypoechoic (more hypoechoic than strap muscles). Shape was defined as wider-than-tall when antero-posterior diameter/transvers diameter <1; taller-than-wide when antero-posterior diameter/transvers diameter >1. Margins were classified as smooth, ill-defined, lobulated or irregular, extrathyroidal extension. When an echogenic foci is identified, it was defined as macrocalcification (if it causes acoustic shadowing), peripheral rim calcification, or microcalcification. Two researchers categorized each nodule with consensus based on ACR-TIRADS, EU-TIRADS, and KTIRADS utilizing the afore mentioned ultrasound findings (Figure 1, Figure 2 and Figure 3).

FNABs were performed under guidance of ultrasound by a radiologist with 13 years of experience in thyroid radiology and interventional radiology. Biopsies were conducted using an aspiration technique and a 27G needle, with samples taken from two distinct locations of the nodules. A pathologist with 15 years of experience in thyroid cytopathology analyzed direct smears of each specimen and classified them according to the Bethesda system 2017 for each nodule. The reference standard diagnosis (malignant or benign) was determined by the histopathological examination of the resected nodule in cases where surgery was performed. When the nodule was managed without surgery, FNAB cytology was used as the reference standard. Nodules were classified as malignant if they were categorized as Bethesda class V and VI, and benign when they were categorized as Bethesda class II. All nodules that were cytologically classified as Bethesda classes I, III, and IV but had no histologic diagnoses were excluded from the final evaluation, unless a repeat FNAB had produced definitive results.

Statistical analysis was conducted with IBM SPSS software (version 23). Continuous variables were summarized using the mean with standard deviation. Categorical variables were summarized using frequencies or percentages. The Chi-square test was employed to compare categorical variables. Sensitivity, specificity, false positive rate (FPR), false negative rate (FNR), predictive values, and likelihood ratios were used to investigate the diagnostic accuracy of each of the three guidelines. The Mann–Whitney U test or Fisher’s exact test was utilized to compare continuous variables between benign and malignant nodules. McNemar’s test was applied to compare the sensitivity and specificity of the three guidelines. A *p*-value of less than 0.05 was regarded as significant.

## 3. Results

One patient with previously diagnosed papillary carcinoma and 10 nodules (7 with non-diagnostic FNA results; 2 with atypia or follicular lesion of undetermined significance; 1 with suspected follicular lesion) that had no histological diagnoses or a repeated FNAB did not produce definitive results were excluded from the study. A total of 225 thyroid nodules were included in our investigation. Among the 225 thyroid nodules (44 from males and 181 from females), 52 were malignant and 173 were benign, as determined by cytology or histopathological evaluation. Final diagnosis of all malignant nodules and 15 benign nodules were based on histopathological examination, whereas 158 benign nodules were confirmed with cytological analysis. The mean age of the study population was 47.10 ± 11.4. The mean age of patients with benign nodules was 47.68 ± 11.6 years, while the mean age of patients with malignant nodules was 45.17 ± 10.8 years. The demographic characteristics of the study population and nodule size are analyzed in Table 1.

The US features of benign and malignant nodules are presented in Table 2. A significant statistical difference was seen in the sonographic characteristics between benign and malignant nodules (*p* < 0.05). US characteristics such as very hypoechoic echogenicity (80%), taller than wide shape (70.37%), extrathyroidal extension, lobulated or irregular margins (54.54%), rim calcification (62.5%) and microcalcifications (68.42%) were more common in malignant nodules.

ACR-TIRADS exhibits the highest diagnosis accuracy, with a value of 63.1%, among three distinct TIRADS systems. Additionally, the maximum specificity (58.7%) and positive predictive value (36.3%) were achieved with ACR-TIRADS. K-TIRADS had the highest sensitivity (94.2%), negative predictive value (96.1%), and the best negative likelihood ratio (0.13) (Table 3). The number of malignant nodules recommended for FNAB was highest with K-TIRADS (*n* = 49) (Table 4); however, the positive predictive value was lowest with K-TIRADS (33.1%) due to high number of negative FNABs (Table 3). In the majority of parameters, the success of EU-TIRADS was between the other two guidelines with a sensitivity of 90.4%, specificity of 48.6% and diagnostic accuracy of 58.2%. The false positive rate was lowest with ACR-TIRADS (41.6%), followed by EU-TIRADS (51.4%) and K-TIRADS (57.2%) (Table 3). A statistically significant difference in specificity was seen among the guidelines, with the ACR exhibiting the highest specificity (*p* < 0.05 between all three guidelines). Despite a significant difference in sensitivities between ACR and K-TIRADS (*p* < 0.05), no statistically significant difference was seen in the sensitivity of ACR compared to EU-TIRADS, nor between EU-TIRADS and K-TIRADS (*p* = 0.07 and *p* = 0.5, respectively).

## 4. Discussion

Numerous research organizations globally have developed algorithms to improve diagnostic performance in thyroid nodule characterization by a combination of ultrasound features. The present study is limited to the most recently established malignancy stratification systems, specifically ACR, EU and K-TIRADS, due to the considerable number of varied US-based risk stratification algorithms. Furthermore, the EU and K-TIRADS system was chosen for its comparative ease of use, while the ACR-TIRADS was favored for its distinctive methodology grounded in comprehensive point-scoring. The ACR-TIRADS evaluates ultrasound features based on their malignancy risks by assigning scores from 0 to 3, scores are given for five separate ultrasonography characteristics, and their total determines the nodule’s risk classification [8]. This method may be seen as extremely time-consuming for routine application. EU and K-TIRADS employ a more direct methodology by categorizing certain ultrasound characteristics as indicative of a high malignancy risk. According to EU-TIRADS, high-risk nodules are defined by the presence of at least one high-risk characteristic, which includes a taller-than-wide nodule shape, irregular margins, microcalcifications, and marked hypoechogenicity [22]. Similarly, a highly suspicious nodule is described by K-TIRADS as any hypoechoic solid nodule with any suspicious US feature of non-parallel orientation, irregular margin or punctate echogenic foci [21]. These three algorithms also demonstrate variances in their concepts. For example, macrocalcification and rim calcification are not accounted for in the EU and K-TIRADS, whereas they contribute additional points to the malignancy risk assessment of a nodule in the ACR-TIRADS. In our investigation, ACR TIRADS revealed the highest number of TR-4 nodules (79 nodules) in comparison to EU and K-TIRADS. This was attributable to macrocalcifications and rim calcifications, which are not recognized as criterion by EU- or K-TIRADS, resulting in the elevation of a solid isoechoic nodule from category TR-3 to category TR-4 (Table 5).

In the present study, most of the patients who underwent FNAB were female. While malignancy rates were slightly elevated in males compared to females, consistent with the findings of Shen et al. [26], Hekimsoy et al. [27] and Özdemir et al. [28], no statistically significant difference between males and females was seen (Table 1). In line with the findings of Kamran et al. [29] and Frates et al. [30], no significant difference was seen between the size of malignant and benign nodules (Table 2). In accordance with previous studies, very hypoechoic echogenicity, taller-than-wide shape, lobulated or irregular margins, extrathyroidal extension and existence of punctate echogenic foci were found to be more prevalent in malignant nodules in our series [9,28,31,32,33,34]. In current study, we found that five (62.5%) of eight nodules with rim calcification were malignant, which is not compatible with the previous studies (Table 2) [32,35].

According to the findings in our study, the most sensitive guideline was K-TIRADS (94.2%). It was slightly higher than that of EU-TIRADS (90.4%), but significantly distinct from ACR-TIRADS (78.8%). On the other hand, K-TIRADS also obtained the lowest levels of specificity in our study, which resulted in an increase in the rate of unnecessary biopsies (false positive rate) for this guideline. We think that the higher sensitivity of K-TIRADS primarily results from less size cutoff values of this guideline especially for intermediate suspicious (TIRADS-4) nodules. This situation, on one hand, reduces the number of undiagnosed malignancies, while on the other hand, leads to a decrease in specificity. The nodular size criteria for FNAB recommendations vary among these three algorithms, particularly for “mildly suspicious” (TIRADS-3) and “moderate suspicious” (TIRADS-4) nodules. The EU and K-TIRADS guidelines recommend FNAB for “mildly suspicious” nodules above 2.0 cm in diameter, whereas ACR-TIRADS sets the threshold at 2.5 cm for such nodules. Similarly, for “moderate suspicious” nodules K-TIRADS recommends a size threshold for FNAB within the range of 1.0 cm and 1.5 cm based on ultrasound features, nodule location, clinical risk factors and patient factors (age, comorbidities, and preferences), while ACR and EU-TIRADS have a size threshold of 1.5 cm in diameter for FNAB of these nodules (Table 5). ACR-TIRADS had the highest overall diagnostic accuracy, specificity and positive predictive value, reducing the number of unnecessary FNABs that may potentially result in socioeconomic advantage. A proportionate result was achieved by EU-TIRADS, as its statistics were between the other two guidelines in nearly all parameters. In this regard, our results are comparable with the results of previous studies (Table 6). In contrast to our findings, Özdemir et al. [28], in their 2025 publication, reported that the sensitivity of K-TIRADS was lower than that of ACR-TIRADS and EU-TIRADS (Table 6). We think this difference can be explained by variations in the study population, as well as the higher proportion of benign nodules in their study.

Performing FNABs without clear indication leads to increased healthcare costs and resource utilization. The strict implementation of the guidelines provided by each of the three TIRADS systems would have significantly decreased the number of FNABs conducted (Table 3). The most significant reduction in our study would have been achieved with ACR-TIRADS in our study with 112 FNABs (49.7%), followed by EU and K-TIRADS with 89 (39.5%) and 77 (34.2%) FNABs, respectively. This can be a major strength of the ACR-TIRADS system with the potential to yield socioeconomic benefits. The results of our study are compatible with the study by Grani et al. [9] who reported this value as 53.4%, 30.7% and 17.1% for the ACR, EU- and K-TIRADS, respectively. Xu et al. also reported this value as 62.6% for ACR-TIRADS and 54.6% for EU-TIRADS in their study published in 2019 [32]. We think that the high number of avoided biopsies by ACR-TIRADS results from the higher size thresholds it establishes for proposing FNAB of nodules categorized as low risk. While the ACR-TIRADS guideline reduces the rate of benign nodules undergoing FNAB, it may also lead to a decrease in the percentage of malignant nodules undergoing FNAB. This is unavoidable due to the existence of certain malignancies that exhibit benign sonographic characteristics. In the study by Hekimsoy et al. [27], the malignancy rates among nodules with deferrable biopsies were reported as 16% for ACR-TIRADS and 15% for EU-TIRADS guidelines. In the current study, the malignancy rates of deferrable FNABs were 9.8% for ACR-TIRADS, 5.6% for EU-TIRADS, and 3.8% for K-TIRADS. Grani et al. [9] revealed better outcomes with the ACR-TIRADS guideline than those of EU- and K-TIRADS in malignancy rates of deferrable FNABs: 2.2%, 3.2% and 3.5%, respectively. Xu et al. [32] identified significantly elevated malignancy rates in nodules for which FNAB was deferrable according to the ACR and EU-TIRADS guidelines: 33.1% and 37.7%, respectively. These variations are likely attributable to differences in the prevalence of malignant nodules across the studies, which were reported as 7.2% by Grani et al. [9], 40% by Xu et al. [32], 24.7% by Hekimsoy et al. [27] and 23.1% in our study.

In our investigation, eleven malignant nodules have been missed by ACR-TIRADS, five by EU-TIRADS, and three by K-TIRADS because they did not meet the FNAB size criteria (Table 7). The most missed malignancy pattern by ACR-TIRADS is “solid hyperechoic” nodules between 1.0 and 1.5 cm in diameter. According to the ACR-TIRADS classification, solid hyperechoic nodules receive three points, categorizing them as TR-3 and any additional high-risk ultrasound features, such as microcalcification, irregular margins, or a taller-than-wide shape, do not elevate the nodule’s category to TR-5 on their own. The FNAB threshold for the TR-4 category is 1.5 cm, as per the ACR-TIRADS guidelines. We believe that is the primary explanation for the elevated false negative rate of the ACR-TIRADS guideline in comparison to the other two guidelines. These nodules are classified as EU-TIRADS-5 and K-TIRADS-4 according to the corresponding criteria and enable cytologic diagnosis with a FNAB threshold of 1.0 cm in both categories. One “solid hyperechoic” nodule that met the FNAB criteria of ACR-TIRADS guideline was missed by other two guidelines due to rim calcification that receives two points and elevates the category to TR-4 as per ACR-TIRADS. Rim calcification is not assessed within the EU and K-TIRADS classification. Missing malignant nodules could lead to a delay in diagnosis, allowing cancer to progress to a more advanced stage. Some of these nodules may spread to lymph nodes or distant organs if left undiagnosed, reducing treatment success rates. A delayed diagnosis may necessitate more extensive surgical procedures. However, the malignant nodules missed by the three guidelines were still advised for follow-up and could have ultimately been discovered if there had been an increase in size during follow-up. According to the ACR-TIRADS guideline, follow-up sonograms are recommended every year for up to 5 years for TR5 nodules, at 1, 2, 3, and 5 years for TR4 nodules, and at 1, 3, and 5 years for TR3 lesions [8]. As per the EU-TIRADS guideline, follow-up ultrasonography is recommended every 6–12 months for EU-TIRADS 5 nodules, at the end of one year for EU-TIRADS 4 nodules, and between 3 to 5 years for EU-TIRADS 3 nodules [22]. According to the K-TIRADS guideline, US scans are recommended every 6 months for the first 1–2 years for K-TIRADS 5 nodules, followed by annual scans, while for K-TIRADS 3 and 4 nodules, follow-up is recommended at 1, 3, and 5 years [21].

Our study contains various limitations that must be acknowledged when interpreting our findings. The main limitation of our study is its single-center design. Another limitation of our study was the lack of histopathological diagnoses for all nodules. All nodules with malignant cytological results underwent surgery that enables us to reach histopathological diagnoses but only two nodules with benign cytology underwent surgery due to clinician’s discretion and patient’s preference. Cytology results were used as reference standard diagnosis in remaining nodules. Also, inter-observer and intra-observer variability in this study were not assessed. On the other hand, a key strength of our study is its prospective design: the ultrasound characteristics of each nodule were assessed during real-time examinations conducted prior to performing FNABs. In this context, each of the three internationally recognized TIRADS approaches we evaluated identified several thyroid nodules for which FNAB requests were likely unnecessary.

## 5. Conclusions

The comparison of the three guidelines in our study indicated that they serve as excellent screening strategies for identifying malignant thyroid nodules, exhibiting high sensitivity and a low negative likelihood ratio. This study also established that ACR-TIRADS, K-TIRADS and EU-TIRADS had good diagnostic accuracy, and they can serve as effective tools for the management of patients with thyroid nodules in routine clinical practice, but they have their own advantages and disadvantages. While ACR-TIRADS stands out with its high specificity values, EU- and K-TIRADS are notable for their high sensitivity values. In this sense, the characteristics of the population may determine which guideline should be applied. For example, EU- and K-TIRADS might be better options in populations with a high prevalence of thyroid malignancies, while ACR-TIRADS could be preferred in situations where a cost-effective approach is adopted. In this regard, we believe that further studies incorporating differences in characteristics of populations should be conducted.

## Figures and Tables

**Table 1:** The demographic features of study population and size of thyroid nodules.

	FNAB Results			
Parameter	Benign	Malignant	Total	*p* Value
Number of patients	173 (76.9%)	52 (23.1%)	225	
Gender				0.07
Male	26 (66.7%)	13 (33.3%)	44	
Female	147 (79%)	39 (21%)	181	
Patient age (years), mean ± SD	47.68 ± 11.6	45.17 ± 10.8		0.321
Nodule size (mm), mean ± SD	20.02 ± 7.5	17.8 ± 5.6		0.098

FNAB, fine needle aspiration biopsy. SD, standard deviation.

**Table 2:** Comparison of sonographic characteristics between benign and malignant thyroid nodules.

	FNAB Results			
Parameter	Benign *n* (%)	Malignant *n* (%)	Total	*p* Value
Composition				0.027
Cystic or almost completely cystic	0	0	0	
Spongiform	1 (100)	0	1	
Mixed cystic and solid	27 (96.43)	1 (4.57)	28	
Solid or almost completely solid	145 (73.98)	51 (26.02)	196	
Echogenicity				0.000
Anechoic	0	0	0	
Hyperechoic or isoechoic	131 (83.43)	26 (16.57)	157	
Hypoechoic	41 (65.08)	22 (34.92)	63	
Very hypoechoic	1 (20)	4 (80)	5	
Shape				0.000
Wider-than-tall	165 (83.33)	33 (16.66)	198	
Taller-than-wide	8 (29.62)	19 (70.37)	27	
Margins				0.000
Smooth	156 (81.67)	35 (18.32)	191	
Ill defined	7 (63.63)	4 (36.36)	11	
Lobulated or irregular	10 (45.45)	12 (54.54)	22	
Extrathyroidal-extension	0	1 (100)	1	
Calcifications				0.000
None or comet-tail artifacts	129 (83.76)	25 (16.23)	154	
Macrocalcifications	35 (79.54)	9 (20.45)	44	
Rim calcification	3(37.5)	5 (62.5)	8	
Microcalcification	6 (31.57)	13 (68.42)	19	

FNAB, fine needle aspiration biopsy.

**Table 3:** The comparison of three TIRADS guidelines in terms of diagnostic value.

	Diagnostic Accuracy	FNR	FPR	Sensitivity (95% CI)	Specificity (95% CI)	PPV (95% CI)	NPV (95% CI)	PLR (95% CI)	NLR (95% CI)	No. of Avoided Biopsies (%)
ACR-TIRADS	63.1%	21.2%	41.6%	78.8 (66.5–88.4)	58.4 (51–65.6)	36.3 (27.8–45.4)	90.2 (83.8–94.8)	1.89 (1.51–2.37)	0.36 (0.21–0.62)	112 (49.7)
EU-TIRADS	58.2%	9.6%	51.4%	90.4 (80.5–96.4)	48.6 (41.2–56)	34.6 (26.9–42.8)	94.4 (88.3–97.9)	1.75 (1.48–2.08)	0.19 (0.08–0.46)	89 (39.5)
K-TIRADS	54.6%	5.8%	57.2%	94.2 (85.7–98.5)	42.8 (35.5–50.2)	33.1 (25.9–40.9)	96.1 (90.2–99)	1.64 (1.42–1.90)	0.13 (0.04–0.41)	77 (34.2)

ACR, American College of Radiology. EU, European. K, Korean. TIRADS, Thyroid Imaging Reporting and Data System. FNR, false negative rate. FPR, false positive rate. PPV, positive predictive value. NPV, negative predictive value. PLR, positive likelihood ratio. NLR, negative likelihood ratio.

**Table 4:** Comparison of fine needle aspiration biopsy indications with cytologic or histopathological results.

	FNAB Indication	Cytologic or Histopathological Result	
		No. of Benign Nodules	No. of Malignant Nodules
ACR-TIRADS	+	72	41
	−	101	11
EU-TIRADS	+	89	47
	−	84	5
K-TIRADS	+	99	49
	−	74	3

FNAB, fine needle aspiration biopsy.

**Table 5:** Comparison of ultrasonography and FNAB approaches of three TIRADS guidelines.

Feature	ACR-TIRADS	EU-TIRADS	K-TIRADS
Basic Approach	Point-based system (scored based on 5 features)	Category-based classification	Category-based classification
Categories	TR1–TR5 (1: Benign, 5: High risk)	EU-TIRADS 1–5 (1: No nodule, 5: High risk)	K-TIRADS 1–5 (1: No nodule, 5: High risk)
Composition	Cystic ^¶^, spongiform ^¶^, mixed ^¥^, solid ^Ͳ^	Cystic, spongiform, solid	Cystic, spongiform, partially cystic, solid
Echogenicity	Anechoic ^¶^, hyper- or isoechoic ^¥^, hypoechoic ^Ͳ^, very hypoechoic ^β^	Anechoic, hyperechoic, mildly hypoechoic, marked hypoechogenicity	Anechoic, hyper- or isoechoic, hypoechoic
Shape	Wider-than-tall^¶^, taller-than-wide ^β^	Oval or taller-than-wide	Oval or taller-than-wide
Margin	Smooth ^¶^, ill-defined ^¶^, lobulated or irregular ^Ͳ^, extrathyroidal extension ^β^	Smooth, irregular	Smooth, irregular
Echogenic Foci	None ^¶^, macrocalcification ^¥^, rim calcification ^Ͳ^, microcalcification ^β^	None, microcalcification	None, punctate echogenic foci
Risk Stratification	TR3: 3 points TR4: 4–6 points TR5: ≥7 points	EU-TIRADS 3: Iso-hyperechoic, solid with no high-risk features. EU-TIRADS 4: Mildly hypoechoic with no high-risk features. EU-TIRADS 5: With at least one high-risk features *	K-TIRADS 3: Partially cystic or hyper-/isoechoic nodule without suspicious features K-TIRADS 4: Solid hypoechoic nodule without suspicious features, partially cystic or hyper-/isoechoic nodule with any of the suspicious features ^α^, entirely calcified nodules. K-TIRADS 5: Solid hypoechoic nodule with any suspicious features ^α^
FNAB Indication	TR3 (≥2.5 cm), TR4 (≥1.5 cm), TR5 (≥1 cm)	EU-TIRADS 3 (>20 mm), EU-TIRADS 4 (>15 mm), EU-TIRADS 5 (>10 mm)	K-TIRADS 3 (>2 cm), K-TIRADS 4 (>1–1.5 cm), K-TIRADS 5 (>1 cm)

FNAB, fine needle aspiration biopsy. ^¶^ Assign 0 points, ^¥^ Assign 1 point, ^Ͳ^ Assign 2 points, ^β^ Assign 3 points. * Irregular shape, irregular margin, microcalcification, marked hypoechogenicity. ^α^ Punctate echogenic foci, non-parallel orientation (taller than wide), and irregular margins.

**Table 6:** Prior studies that investigate sensitivity and specificity of ACR, EU and K-TIRADS in distinguishing benign and malignant thyroid nodules.

Study	ACR-TIRADS		EU-TIRADS		K-TIRADS	
	Sensitivity %	Specificity %	Sensitivity %	Specificity %	Sensitivity %	Specificity %
Mohan et al. [35]	93.26	50.75	95.51	26.68	97.75	23.51
Grani et al. [9]	83.3	56.2	86.1	32	91.7	17.8
Yoon et al. [36]	77.3	67.7	87.4	38.9	95.7	23.6
Tan et al. [37]	85.7	51.1	57.1	83.2	100	40.2
Ha et al. [25]	76.1	61.8	84.6	39.3	91	39.7
Özdemir et al. [28]	84	87	91.7	48.5	71.4	44.4

ACR, American College of Radiology. EU, European., K, Korean. TIRADS, Thyroid Imaging Reporting and Data System.

**Table 7:** Ultrasound features of 12 malignant nodules missed by guidelines.

	Size	Composition	Echogenicity	Shape	Margin	Echogenic Foci	TIRADS System		
							ACR	EU	K
1	13 mm	Solid	Hyperechoic	W > T	Smooth	Microcal.	4 ^q^	5	4
2	12 mm	Solid	Hyperechoic	W > T	Smooth	Microcal.	4 ^q^	5	4
3	13 mm	Solid	Hypoechoic	W > T	Smooth	None	4 ^q^	4 ^q^	4
4	14 mm	Solid	Hyperechoic	T > W	Smooth	None	4 ^q^	5	4
5	24 mm	Solid	Hyperechoic	W > T	Smooth	None	3 ^q^	3	3
6	14 mm	Solid	Hyperechoic	W > T	Smooth	Microcal.	4 ^q^	5	4
7	17 mm	Solid	Hyperechoic	W > T	Smooth	Rim	4	3 ^q^	3 ^q^
8	12 mm	Solid	Hypoechoic	W > T	Smooth	None	4 ^q^	4 ^q^	4
9	15 mm	Solid	Hyperechoic	W > T	Smooth	None	3 ^q^	3 ^q^	3 ^q^
10	18 mm	Solid	Hyperechoic	W > T	Smooth	None	3 ^q^	3 ^q^	3 ^q^
11	14 mm	Solid	Hypoechoic	W > T	Irregular	None	4 ^q^	5	5
12	12 mm	Solid	Hyperechoic	W > T	Smooth	Microcal.	4 ^q^	5	4

Microcal., microcalcification. W, wide. T, tall. ^q^, represents the missed malignancy.

## Data Availability

The data presented in this study are available on request from the corresponding author. The data are not publicly available due to privacy and ethical reasons.
